# Evaluation of the Effect of Sampling Time on Biomarkers of Stress, Immune System, Redox Status and Other Biochemistry Analytes in Saliva of Finishing Pigs

**DOI:** 10.3390/ani12162127

**Published:** 2022-08-19

**Authors:** Alba Ortín-Bustillo, María D. Contreras-Aguilar, Camila P. Rubio, María Botia, José J. Cerón, Marina López-Arjona, Silvia Martínez-Subiela, Damián Escribano, Fernando Tecles

**Affiliations:** 1 Interdisciplinary Laboratory of Clinical Analysis of the University of Murcia (Interlab-UMU), Regional Campus of International Excellence ‘Campus Mare Nostrum’, University of Murcia, Campus de Espinardo s/n, 30100 Murcia, Spain; 2 Department of Animal and Food Science, Universitat Autònoma de Barcelona, 08193 Barcelona, Spain; 3 Department of Animal Production, Regional Campus of International Excellence ‘Campus Mare Nostrum’, University of Murcia, Campus de Espinardo s/n, 30100 Murcia, Spain

**Keywords:** analytes, biomarkers, fattening pigs, hour of the day, saliva

## Abstract

**Simple Summary:**

Saliva is a sample with a high potential in pigs since it is usually easy to obtain and its collection from animals causes less stress than blood sampling. However, the possible effects of daily variations in many salivary biomarkers are still unknown in this species. In our report, the possible variations depending on the sampling time in the day in a panel of 26 salivary biomarkers related to stress, immune system, redox status and other physiological functions in the saliva of pigs were evaluated. In our experimental conditions, daily variations were observed in cortisol, α-amylase, total esterase, butyrylcholinesterase, lipase, adenosine deaminase isoenzyme 1, uric acid, superoxide dismutase, alanine aminotransferase, aspartate aminotransferase, alkaline phosphatase, creatine kinase, lactate dehydrogenase, triglycerides and lactate. In some analytes, these differences appeared in both sexes, whereas others only showed differences in one sex. These variations should be taken into consideration for an appropriate interpretation of these analytes in the saliva of healthy pigs.

**Abstract:**

This study aims to evaluate the possible variations due to the sampling time in the day in 26 analytes of pigs’ saliva, related to stress, the immune system, redox status and other biomarkers related to metabolism and selected tissues and organs, in order to know the possible effects of the hour of the day in their interpretation. These analytes were measured in saliva obtained from a population of 40 clinically healthy pigs from 8 a.m. to 8 p.m., every 4 h in the same day. In our experimental conditions, daily variations were observed in cortisol, salivary α-amylase, total esterase activity, butyrylcholinesterase, lipase, adenosine deaminase isoenzyme 1, uric acid, superoxide dismutase, alanine aminotransferase, aspartate aminotransferase, alkaline phosphatase, creatine kinase, lactate dehydrogenase, lactate and triglycerides. These changes appeared in both sexes, except for adenosine deaminase isoenzyme 1 and superoxide dismutase which only showed differences in females. In conclusion, this report indicates that, in the experimental conditions of this trial, the time of the day and sex can influence the values obtained in various salivary analytes in pigs. These variations should thus be taken into consideration for an adequate interpretation of these analytes when used for the evaluation of health and welfare in this species.

## 1. Introduction

Saliva is gaining attention and interest in veterinary science and, in particular, in pig production as a useful sample to evaluate different biomarkers related to health and welfare. This is because its collection is much easier than blood and does not induce animal stress or discomfort [1]. In pigs, a comprehensive panel of analytes including biomarkers of stress, inflammation, immune system, redox status and other analytes related to different physiological aspects and various tissues and organs, such as hepatic and muscle integrity, can currently be measured in this fluid [2]. This is included under the concept of sialochemistry [3,4] which has previously been applied to evaluate changes in analytes during pregnancy, farrowing, lactation [5] and fattening [6] in pigs and also in other species such as horses and cows [7,8].

The knowledge of the possible influence of the sample collection time during the day in salivary analytes is of high importance for an adequate interpretation of the biomarkers [9]. For example, in healthy horses, the values found at certain times of the day for some salivary analytes such as total esterase activity (TEA) or butyrylcholinesterase (BChE) reached those that were found in psychological acute stress situations or diseases [10]. In pigs, cortisol or immunoglobulin A (IgA) showed significant changes in saliva depending on the hour of the day [11,12,13]. However, no differences have been found in certain salivary analytes, such as chromogranin A [14]. In addition, C-reactive protein and haptoglobin can be measured in the saliva of pigs at any time of the day without significant differences [15].

Despite these previous reports, to our knowledge there are no studies about the variations depending on the hour of the day in other salivary biomarkers of stress different than cortisol, such as α-amylase (sAA), TEA, BChE or lipase (Lip). In addition, no studies about this topic were found for adenosine deaminase (ADA), which is a biomarker of the immune system in pigs [16], for biomarkers of redox status that can be measured in pigs [17], and other analytes included in the sialochemistry profile [2].

Therefore, this study aims to evaluate: (1) if the values from a panel of salivary analytes can change when measured at different times of the day in pigs; and (2) if these changes could be related to sex. This panel will be integrated by a total of 26 analytes including biomarkers of stress such as cortisol, sAA, TEA, BChE, and Lip; biomarkers of immune system such as ADA; biomarkers related to redox status such as the cupric reducing antioxidant capacity (CUPRAC), the Trolox equivalent antioxidant capacity (TEAC), the ferric reducing ability of saliva (FRAS), superoxide dismutase (SOD), uric acid (UA), hydrogen peroxide (peroxidase activity Pox-Act), reactive oxygen-derived compounds (d-ROMs) and the advanced oxidation protein products (AOPP); and other analytes related to metabolism and physiological status of different tissue and organs such as alanine aminotransferase (ALT), aspartate aminotransferase (AST), alkaline phosphatase (ALP), γ-glutamine transferase (GGT), lactate dehydrogenase (LDH), creatine kinase (CK), urea, creatinine, triglycerides, total protein, glucose, lactate, calcium and phosphorus.

## 2. Materials and Methods

### 2.1. Animals

The experiment was conducted on a commercial farm in the southeast of Spain in May 2022. All pigs included in the study were LargeWhite. A total of 40 animals were randomly selected for inclusion in the experimental procedure. They were 20 males and 20 females with an age of 5 months and 1 week, approximately. Animals were kept under general commercial housing and conditions conforming to European Union guidelines (Directive 2010/63/EU1), and had ad libitum access to a nutritionally balanced diet and water.

Saliva was sampled from the pigs at 0800 h, 1200 h, 1600 h and 2000 h (8 a.m., 12 a.m., 4 p.m. and 8 p.m., respectively) within a same day. The temperature of the pens at these sampling time-points varied from 19 °C in the morning to 24 °C at midday. Saliva was collected using Salivette tubes (Salivette, Sarstedt, Aktiengesellschaft & Co, Nümbrecht, Germany) as reported previously [6]. Pigs were allowed to chew a sponge attached to a flexible thin metal rod for 1 min. Each sponge was then placed individually in the Salivette tubes and stored on ice until arrival at the laboratory (less than 1 h). At the laboratory, the tubes were centrifuged at 3000× *g* for 10 min. Saliva samples were transferred into 1.5-mL Eppendorf tubes and frozen at −80 °C for the analysis.

### 2.2. Stress Biomarkers

All the assays used for stress biomarkers have been previously validated in porcine saliva. Salivary cortisol was measured by an in-house indirect competitive AlphaLisa method [18]. sAA activity was measured by an spectrophotometric assay (a-Amylase, OSR6182, Beckman Coulter Inc., Fullerton, CA, USA) [19]. TEA was analysed by an automated assay using 4-nitrophenyl acetate (4-NA) as substrate [20]. BChE was measured with 5,5′-dithiobis-2-nitrobenzoic acid (DTNB, Sigma-Aldrich, Merck KGaA, Darmstadt, Germany; final concentration, 0.29 mM) as chromophore and butyrylthiocholine iodide (BTCI, Sigma-Aldrich; final concentration, 16.18 mM) as substrate [21]. Lip was determined by a colorimetric assay (Lipase, Beckman Coulter, Brea, CA, USA). Salivary sAA, BChE and Lip were measured in an automated analyser (Olympus AU600, Olympus Diagnostica GmbH, Beckman Coulter).

### 2.3. Immune System Biomarkers

ADA activity was measured by a kinetic spectrophotometric assay (Adenosine Deaminase assay kit, Diazyme Laboratories, Poway, CA, USA), validated in pigs [22]. Total ADA and ADA2 isoenzyme were measured, respectively, in absence and presence of the specific ADA1 inhibitor erythro-9-(2-hydroxy-3-nonyl) adenine (EHNA, Sigma-Aldrich, City of Saint Louis, MO, USA) in the reaction media at a final concentration of 0.47 mM, as previously described [16]. The isoenzyme ADA1 was calculated from the difference between total ADA and ADA2. ADA assays were carried out in the Olympus AU600 analyser.

### 2.4. Redox Biomarkers

CUPRAC was determined based in the reduction of Cu^2+^ to Cu^1+^ by the non-enzymatic antioxidants of the specimen [23]. TEAC was measured by the reduction of 2,20-azino-bis(3 ethylbenzothiazoline-6-sulfonic acid) radical [24]. FRAS was determined by the reduction of the ferric-tripyridyltriazine (Fe^3+^-TPTZ) complex [25]. UA was measured by a commercially available colorimetric assay (Beckman Coulter). SOD was measured by a commercially available assay (SOD, Randox Laboratories Ltd., Crumlin, UK). The Pox-Act determination was based on the assay described by Tatzber et al. in which the oxidation of 3,5,3′,5′-Tetramethylbenzidine (TMB) by peroxides in the sample is monitored. The results were also expressed in µmol of peroxide hydrogen per L of the sample (µmol/L) [26]. Salivary d-ROMs levels were determined based on monitoring the N,N-Diethyl-p-phenylenediamine radical cation concentration as previously described with results expressed in Carratelli units (Carr units) [27]. AOPP were assessed based on a previous reported assay [28]. All the assays used for redox biomarkers measurements were performed in an automated analyser (Olympus AU600), and had been validated in saliva of pigs [17].

### 2.5. Analytes Related with Metabolism and Different Tissues and Organs

AST, ALP, GGT, LDH, CK, urea, creatinine, triglycerides, glucose, lactate, calcium and phosphorous were measured by spectrophotometric methods (Beckman Coulter). Total protein (TP) was analysed by an assay for urinary proteins quantification (Protein in Urine and CSF, Spinreact, Barcelona, Spain). All the assays showed an imprecision lower than 15%, were linear after serial sample dilution and were performed in an Olympus AU600 biochemistry autoanalyzer.

### 2.6. Statistical Analysis

A Shapiro–Wilk test was used to evaluate data distribution. All analytes showed a non-normal distribution. Therefore, they were log-transformed by applying the formula ln x = ln(x + 1) for further analysis [10]. After transformation, significant daily changes in the salivary biomarkers measured were evaluated by a mixed linear model of repeated measures considering fixed factors of time (from 0800 h to 2000 h) and gender, being the individual considered as a random factor. When any of the fixed factors was significant, it was further evaluated with a univariate analysis and Bonferroni post-hoc test. Changes were considered significant when *p* < 0.05. The statistical analyses were calculated using Graph Pad Prism 8 (GraphPad Software, San Diego, CA, USA) and SPSS statistics package (IBM SPSS Statistics for Windows, Version 28.0.1. IBM Corp, Armonk, NY, USA).

## 3. Results

**Figure 1:**
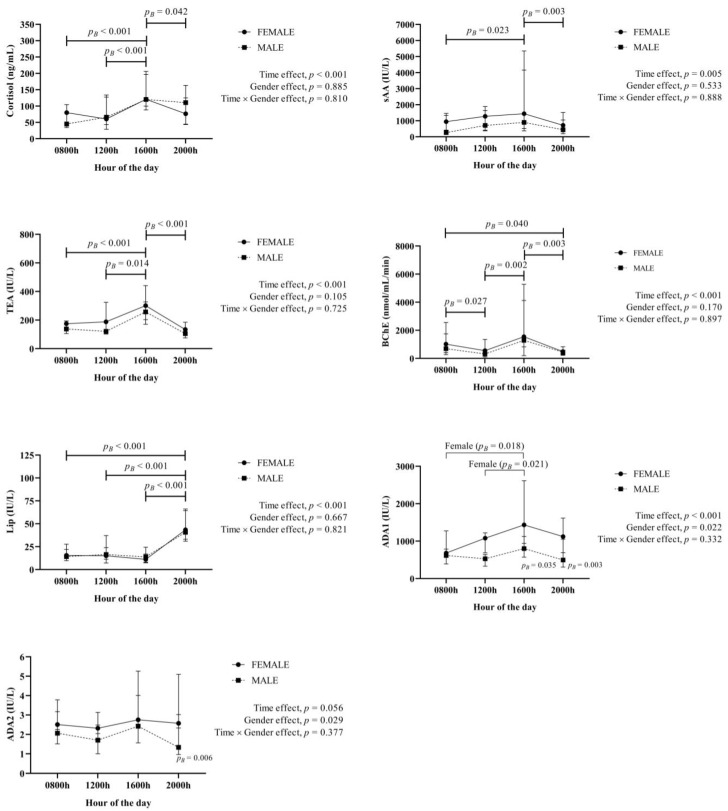
Results for the stress (cortisol, salivary α-amylase (sAA), total esterase activity (TEA), butyrylcholinesterase (BChE) and lipase (Lip)) and immune system (adenosine deaminase (ADA) isoenzymes 1 and 2) biomarkers obtained in saliva from 40 healthy fattening pigs (20 females, 20 males) obtained at different hours of the day. *p_B_*: *p* value of the Bonferroni post-hoc analysis.

The results for the stress and immune system biomarkers appear in Figure 1. Cortisol, sAA, TEA, BChE and Lip showed a significant time effect, with an increase at 1600 h for cortisol, sAA, TEA and BChE, whereas Lip showed a significant increase at 2000 h. ADA1 isoenzyme showed significant changes for time and gender fixed effects, with a significant increase at 1600 h only in females. ADA2 isoenzyme values showed a significant gender effect, with higher values at 2000 h in females than in males.

**Figure 2:**
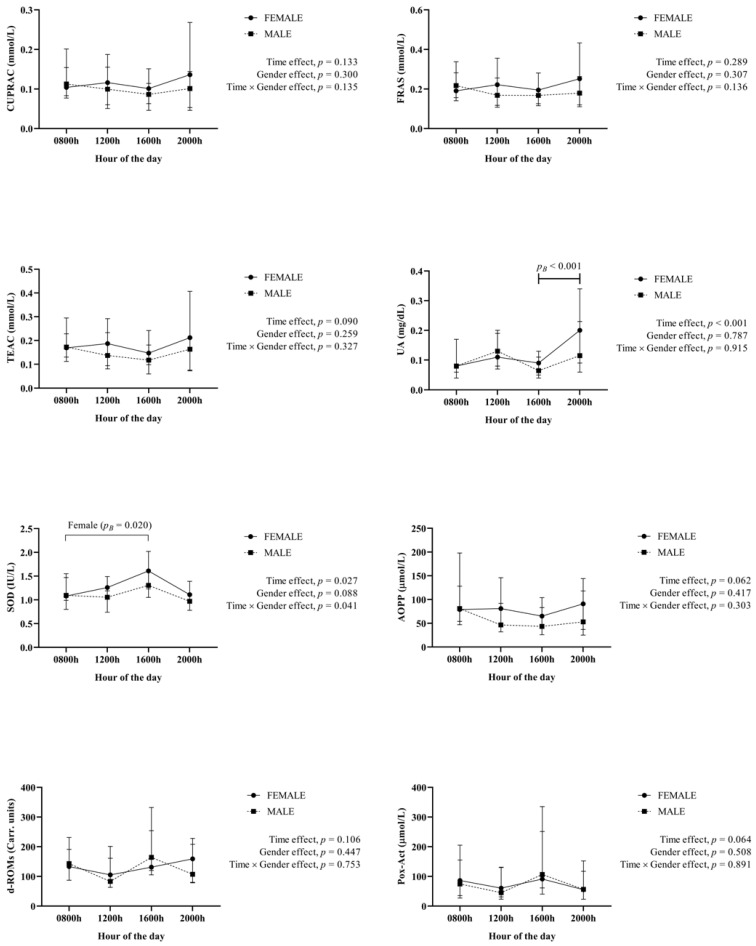
Results for the Redox statuss biomarkers (cupric reducing antioxidant capacity (CUPRAC), the Trolox equivalent antioxidant capacity (TEAC), the ferric reducing ability of saliva (FRAS), uric acid (UA), superoxide dismutase (SOD), the advanced oxidation protein products (AOPP), reactive oxygen-derived compounds (d-ROMs) and hydrogen peroxide (peroxidase activity Pox-Act)) obtained in saliva from 40 healthy fattening pigs (20 females, 20 males) obtained at different hours of the day. *p_B_*: *p* value of the Bonferroni post-hoc analysis.

Results of the Redox status biomarkers are shown in Figure 2. No significant effects were seen in most of the biomarkers between the different hours of the day; with the exception of UA which showed a significant time effect with an elevation at 2000 h, and SOD that showed a significant time and gender interaction with values showing significant increases at 1600 h only in females.

**Figure 3:**
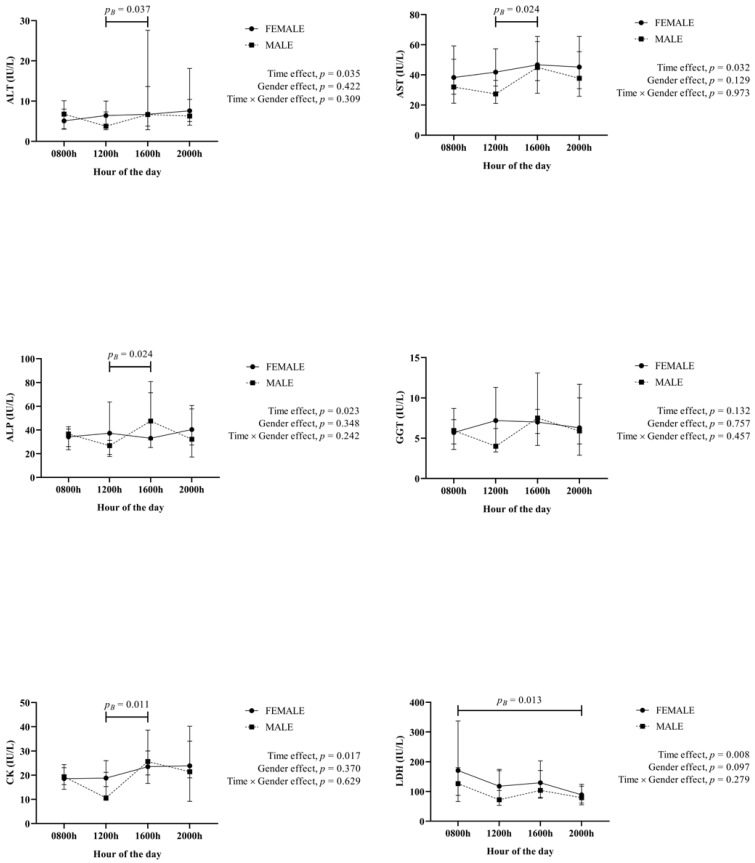
Results for the enzymatic activities (alanine aminotransferase (ALT), aspartate aminotransferase (AST), alkaline phosphatase (ALP), γ-glutamine transferase (GGT), lactate dehydrogenase (LDH) and creatine kinase (CK)) obtained in saliva from 40 healthy fattening pigs (20 females, 20 males) obtained at different hours of the day. *p_B_*: *p* value of the Bonferroni post-hoc analysis.

Results of enzymatic markers related to the physiological status of different tissue and organs are shown in Figure 3. A significant time effect was detected in ALT, AST, ALP and CK, whose values were higher at 1600 h if compared with those obtained at 1200 h, and also for LDH, with higher values at 0800 h if compared with values at 2000 h. No gender effect was observed in any of those enzymatic activities.

**Figure 4:**
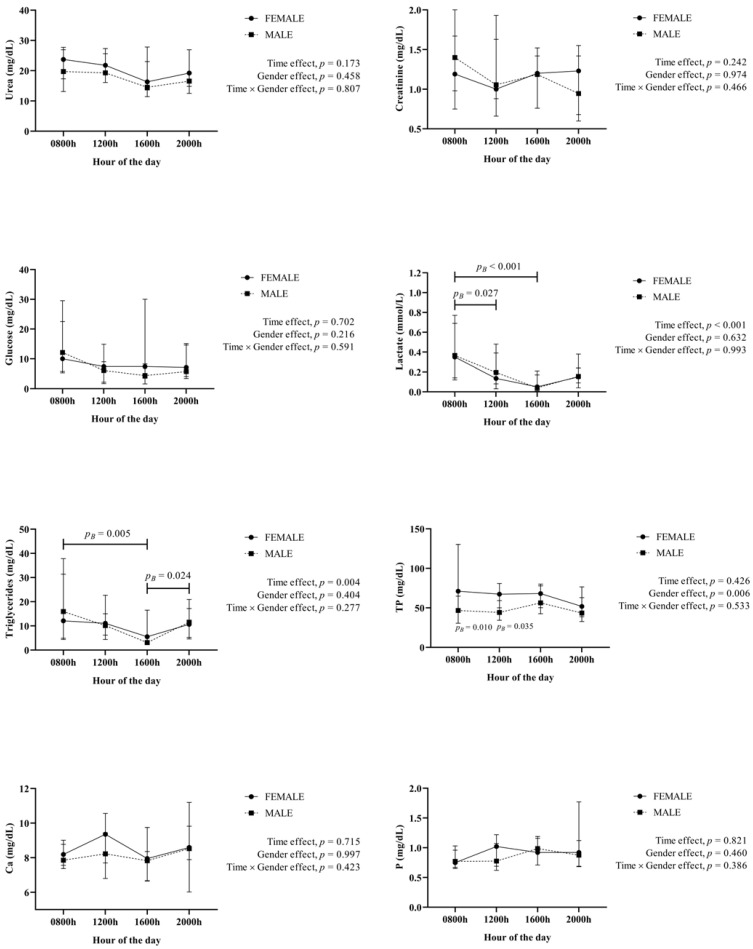
Results for the metabolites (urea, creatinine, glucose, lactate, triglycerides, total protein (TP), calcium (Ca) and phosphorous (P)) obtained in saliva from 40 healthy fattening pigs (20 females, 20 males) obtained at different hours of the day. *p_B_*: *p* value of the Bonferroni post-hoc analysis.

Regarding metabolites (Figure 4), no significant effects were observed for urea, creatinine, glucose, Ca and P. Lactate and triglycerides showed a significant time effect, with lactate values being higher at 0800 h compared to 1200 h and at 1600 h, whereas triglycerides values at 0800 h were lower than at 1600 h.

## 4. Discussion

This study aimed to evaluate if the values of a comprehensive panel of analytes measured in saliva of pigs can vary at different hours of the day and whether or not these differences could depend on the sex. These data could be of practical use in order to select the most appropriate saliva sampling times when measuring analytes for the evaluation of pig health and welfare.

In this report, we performed four different samplings during the day in order to cover the different times in which sampling could be done in routine in farm conditions and according to previous studies made in pigs [13] in which four time points of the day were also selected.

From the 26 analytes measured, to the authors’ knowledge, only cortisol has been previously analysed during different times of the day in saliva of pigs. Similarly to our results, cortisol has been described to peak in porcine saliva at 1600 h in other studies [12,29]. In a report in which pigs were fed two times per day, it was postulated that the increase in salivary cortisol at 1600 h could be explained by increased gluconeogenesis during the fasting periods (11). However, in our case, the pigs were feed ad libitum, so we were not able to determine when the pigs ingested food. In any case, this increase in cortisol at this time has been also described with pigs feeding ad libitum [29] and further studies should be made to elucidate the reason. Overall, the increase of cortisol found in our study at 1600 h, as previously described, supports the validity of our experimental model.

Interestingly, in addition to cortisol, other analytes related to stress such as sAA, TEA and BChE showed a significant increase at 1600 h in our experimental conditions. Although sAA and BChE are more related with the autonomous nervous system [21], whereas cortisol is related with the hypothalamus-hypophysis axis, maybe there could be some mechanism that could potentially activate both axes at this time of the day, and lead to the increase in these analytes.

ADA1 isoenzyme showed a significant increase at 1600 h in females. In human erythrocytes, higher values of ADA were found in samples obtained in the afternoon in comparison with the morning [30]. The reason for this change should be elucidated, and possibly it could have relation with the lymphocyte dynamics, since ADA is an enzyme related with lymphocyte function [31]. In this line, in humans, numbers of total lymphocytes, T and B cells showed higher levels in the afternoon–night than in the morning [32].

Regarding markers of redox homeostasis, UA was the only analyte showing significant differences in both sexes, which a significant elevation at 2000 h. This would be in line with a previous study in humans reporting that the maximal UA concentration, more than two times greater than the minimal values, were measured during the night [33]. A high antioxidant salivary potential could be necessary during the night, when the concentrations of compounds with oxidant potential seem to be at their maximum [34,35].

In metabolites and markers of different tissues and organs, various enzymes such as ALT, AST, ALP and CK showed higher values at 1600 h. To the authors’ knowledge there are not previous studies about daily variations in these analytes and further research should be performed to evaluate the reasons for these changes. Regarding the possible practical applications of these biomarkers, in other species such as dogs, increases in urea and creatinine in saliva have been described in cases of renal failure [36] and increases in CK in muscle damage [37], with theses analytes showing high correlations between saliva and serum. These are examples indicating that in some situations, saliva could be an alternative to serum as biological sample for the measurement of selected metabolites. However, specific studies in the case of the porcine species should be performed to evaluate this.

The gender of the animals did not affect most of the results, except for ADA isoenzymes and SOD activities, which were significantly higher in females than in males at some of the sampling points. Overall, it could be indicated that in general sex does not have a major influence in the variations of salivary analytes during the day with some selected exceptions such as ADA or SOD.

Some analytes such as sAA and ADA showed large individual differences specially at 1600 h. In the case of salivary analytes, it has been reported that those with lower interindividual variability and the greatest magnitude of change with absence of overlapping between the conditions to be evaluated would be preferred [5]. For those biomarkers showing a high interindividual variability, each animal should ideally act as its own control. In addition, based on the findings of this report it could be added that the absence of variations depending on the hour of the day would be a positive factor for a biomarker, since it could be used in samples collected at any time of the day. This would be the case for ADA2 within the immune system biomarkers, GGT within the enzymatic activity markers, and several of the biomarkers for redox homeostasis, which will have the advantage of a lack of changes along the day. ADA has been proposed as a biomarker of inflammation and pain in pigs, showing increases in lameness and prolapses [38] and also increased in sows after parturition, potentially as a response to the postpartum inflammation [39]. GGT has been proven to be a good pain biomarker in species such as the horse [7] and also increases in sows after parturition [5]. In addition, some of the biomarkers of Redox status studied in the manuscript, such as CUPRAC, FRAS, AOPP, Pox-Act and d-ROMs, showed higher values in pigs with sepsis compared to healthy pigs [40]. It is important to point out that in our experimental conditions, in some cases such as cortisol, BChE, Lip and uric acid, the increases found at their peak hours of the day reached similar values than described in pigs with diseases and clinical signs such as lameness and prolapses [38], and this could influence their practical interpretation.

Some of analytes that were used in this panel were significantly correlated with coefficients of correlation higher than 0.7 (data not shown). For example, urea and creatinine which are biomarkers of renal function were correlated. Furthermore, significant correlations were found between the metabolites glucose, triglycerides and lactate; and between sAA and BChE, which are markers of acute stress [21]. In addition, among the antioxidant markers, FRAS was correlated with TEAC, and the oxidants Pox-Act and d-ROMS were correlated. However, it would be of interest to evaluate how these analytes correlate also in situations of stress and disease.

This report has various limitations. First, it should be indicated that the results reported here have been obtained in our experimental conditions and with our analytical methods, and care should be taken when applied to other conditions or different assays. In addition, it should be considered as a pilot study to evaluate the possibility of the presence of changes in salivary analytes depending on the hour of the day and sex. Therefore, further studies should be made in the future at different times of the year and also with more sampling times, in order to obtain a more complete picture of the changes occurring and refine our findings and their possible applications. Studying the possible differences due to season would be of particular importance, since in humans, for example, differences have been found in ADA in winter versus summer [30]. In addition, the possible variations depending on the breed, age, season and productive conditions should be evaluated. Moreover, studying the possible changes depending on different situations of disease or stress would be of interest. Finally, the establishment of proper reference values considering all these sources of variation would be desirable. For this purpose, further studies involving a higher number of animals, at least 120 in case of non-parametric reference intervals [41], should be performed.

## 5. Conclusions

In our experimental conditions there were changes in the values of selected analytes in saliva of pigs depending on the hour of the day in which the sample is collected and sex. Namely, daily variations were observed in cortisol, sAA, TEA, BChE, Lip, ADA1, UA, SOD, ALT, AST, ALP, CK, LDH, lactate and triglycerides. In some analytes, the differences appeared in both sexes, whereas others only showed differences in one sex. Additional studies should be made to elucidate the mechanisms underlying these changes and evaluate if other possible sources of variation such as breed, age, season or productive condition could influence them. However, based on our results, for the measurement of the analytes that showed changes in our study, it would be recommended to obtain saliva samples in the morning; and if afternoon samples should be used, different reference ranges should be established.

## Data Availability

The data presented in this study are available on request from the corresponding author.
